# Editorial: Metabolic pathways to multiple long-term conditions (multimorbidity): focusing on cardio-metabolic multimorbidity (CMM)

**DOI:** 10.3389/fendo.2026.1782242

**Published:** 2026-01-20

**Authors:** Jaideep C. Menon, Sanghamitra Pati

**Affiliations:** 1 Amrita Institute of Medical Sciences and Research Centre, Amrita Vishwa Vidyapeetham, Kochi, India; 2 Indian Council of Medical Research, New Delhi, India

**Keywords:** cardiometabolic multimorbidity, MAFLD, metabolic disease, morbidity measures, multimorbidity

Multimorbidity is the presence of two or more long-term health conditions, which could be a non-communicable disease, chronic mental condition or even chronic infectious diseases (HIV, Hepatitis B), in an individual. This stands separate from co-morbidity which is any distinct additional entity that has existed or may occur during the clinical course of a patient on therapy for an index disease.

Multimorbidity is an issue that clinicians grapple with, especially given the global demographic transition to an increasingly aging population. The pathways to multimorbidity are often shared setting scope for prevention in the at-risk groups. The Research Topic in multimorbidity focusses on cardiometabolic multimorbidity (CMM), covering the themes of epidemiological patterns across various settings globally; pathophysiology and pathways to multimorbidity and CMM; and the therapeutic aspects including challenges at the community level.

Shen et al. did a cross-sectional analysis based on a sample size of 3,779,756 medical records. A network analysis and community classification were performed to illustrate disease networks and patterns of multimorbidity in the elderly population of Shanghai.

Guo et al. cluster analysed multimorbidity patterns in older adults in Shenzhen, China. They analysed data from the Shenzhen aging-related disease cohort, including 8,911 people aged 60 and above after exclusion of missing and abnormal values. The study found that multimorbidity is prevalent among the older adult in Shenzhen and explored their patterns, and found a high prevalence of cardiometabolic comorbidities, reaching 15.83%, and detailed the distribution of specific comorbidity combinations.

Zhou and Yi studied CMM and frailty in middle-aged and the elderly using data from four international cohorts – HRS, CHARLS, ELSA and SHARE – to examine the correlation between frailty and cardiometabolic diseases (CMD). The used the frailty index w for assessing frailty and statistical analyses were performed as a means of analysing the correlation between the number of cardiometabolic conditions and frailty severity. The study found that as the number of cardiometabolic diseases increased, the frailty index rose significantly, with stroke having the most pronounced impact on frailty.

Gao et al. analysed the association between metabolic-associated fatty liver disease and risk of cardiometabolic multimorbidity from a disease trajectory analysis using the data from the UK Biobank. From a median follow-up of 13.85 years, 4,622 new-onset CMM cases emerged among participants free of CMD at baseline. MAFLD was significantly associated with an increased risk of incident CMM independently elevating the risk of incident CMM, emphasizing the necessity of targeted MAFLD interventions for CMM prevention.

Liu et al. studied 4676 eligible participants from the China Health and Retiremuent Longitudinal Study (CHARLS) database, wherein they looked at the association between hemoglobin glycation index (HGI) and the risk of cardiovascular disease in early-stage cardiovascular-kidney-metabolic syndrome (CKM). From their analysis they concluded that HGI is associated with an elevated risk of CVD in participants with early-stage CKM syndrome. Additionally, they opined that HGI could serve as an independent biomarker for guiding clinical decision-making and managing patient outcomes.

Li et al. studied cardiometabolic multimorbidity (CMM) and the risk of sudden cardiac death (SCD) among geriatric community dwellers using longitudinal EHR-derived data. An analysis of records of 55130 elderly population revealed a rate of CMM of 25.3%. Older adults were categorized into different CMM patterns according to the cardiometabolic disease (CMD) status at baseline. Cox proportional hazard models were used to evaluate associations between CMM and SCD. They concluded the risk of SCD varied by the pattern of CMM, and increased with increasing number of CMM among geriatric community dwellers.

Banerjee and Mani explore the molecular pathways linking adipose tissue expansion to multimorbidity and its heterogenous outcomes. Their review explores key pathways, including inflammation, insulin resistance, adipokine dysregulation, and complement system activation, that link obesity to diabetes, cardiovascular diseases, and metabolic syndrome. They go on to analyse of how these pathways drive two major obesity-related conditions: type 2 diabetes and cardiovascular disease, with particular emphasis on the pathophysiology leading to heart failure.

Ma et al. study the association between peripheral thyroid sensitivity defined by the FT3/FT4 ratio and composite adverse outcome among patients with heart failure. Their single centre prospective cohort of 402 patients of heart failure revealed that maintaining or restoring higher FT3/FT4 levels improve outcomes. They opine that regular monitoring of this ratio, coupled with tailored interventions based on thyroid functional status, could enhance risk stratification and therapeutic decision-making.

Guo and Du look at the osteogenic differentiation in vascular smooth muscles with hyperglycaemic. Sustained hyperglycemia drives VSMCs to undergo a phenotypic transition from contractile state to osteo-/chondrogenic lineages through multiple pathophysiological mechanisms. Specifically, hyperglycemia stimulates metabolic reprogramming. This includes enhancing advanced glycation end products (AGEs), trigger vesicles-mediated mineralization (including matrix/extracellular vesicles), oxidative stress, inflammatory cascades, and an imbalance between autophagy and apoptosis.

Yu et al. study the association of pan-immune-inflammation value (PIV) and atherogenic index of plasma (AIP) with chronic coronary syndrome (CCS) in non-alcoholic fatty liver disease patients (NAFLD). They conclude from their assessment of 459 individual with NAFLD that lnPIV and AIP are independent biomarkers for CCS in NAFLD patients. Eight independent variables were used to construct a nomogram which showed values as a tool for CCS risk stratification and personalized management.

Shen et al. studied the expression profiles and roles of microRNAs in cardiac glucose metabolism and to explore their potential as biomarkers for glucose metabolism disorders in diabetic cardiomyopathy (DCM). Their systematic review helped identified 20 consistently dysregulated miRNAs associated with myocardial glucose metabolism. Six dysregulated miRNAs, including miRNA-199a, let-7, miRNA-21, miRNA-133, miRNA-503 and miRNA-378, have potential as candidate miRNA biomarkers of glycometabolism in the heart.

Zhu et al. explored the role of oral microbiome and metabolic profiling in CVD risk stratification and risk prevention as a part of the Suzhou cardiometabolic health study protocol. Their study introduced oral (tongue coating) microbiota as a metabolic marker for the first time, in combination with multiple metabolic factors, to explore their potential in assessing subclinical target organ damage and optimizing cardiovascular risk stratification, in order to provide a new path for the early identification and intervention of CVD.

He et al. investigated the association of body roundness index (BRI) with the risk of CVD and its components including congestive heart failure (CHF), coronary heart disease (CHD), angina, heart attack, and stroke in patients with cardiometabolic syndrome (CMS). At the same time, we hypothesized that BRI would identify CVD better than BMI or waist circumference (WC). They used logistic regression models to evaluate the relationship between BRI and CVD in patients with CMS, using data from the 2009-2018 National Health and Nutrition Examination Survey (NHANES) datasets.

Lekha et al. explored the challenges healthcare providers (HCPs) face in managing people with multiple-long-term conditions (MLTCs) in a south Indian primary care setting, as a qualitative exploratory study. They conducted 33 in-depth, semi-structured interviews with HCPs in four districts of Kerala, India. The study highlights Our study sub-optimal health system preparedness and highlights the challenges for a transitioning primary care for managing people with MLTCs in one of India’s states with a well-developed healthcare system.

Multimorbidity especially CMM is now a norm in clinical practise, especially in the elderly. Understanding the epidemiology and the patterns of multimorbidity becomes crucial for therapy, prevention and for policy-makers and planners. Pathways to multimorbid conditions are often shared, making its identification crucial for effective prevention. Multimorbidity also needs be assessed with a different therapeutic lens for both medications and in the context of morbidity measures. Certain common patterns of multimorbidity would add to disability weights of more than 1 which represents deaths. It makes a case of assigning commoner dyads and triads a disability weight with multimorbidity rather than for the individual conditions. These, and the pathways to multimorbidity need be explored more deeply so as to ensure a more personalised/customised care and not therapy for individual morbid conditions.

